# 3-Phenyl­isoquinolin-1(2*H*)-one

**DOI:** 10.1107/S1600536809000245

**Published:** 2009-01-08

**Authors:** P. Manivel, Venkatesha R. Hathwar, R. Subashini, P. Nithya, F. Nawaz Khan

**Affiliations:** a Chemistry Division, School of Science and Humanities, VIT University, Vellore 632 014, Tamil Nadu, India; b Solid State and Structural Chemistry Unit, Indian Institute of Science, Bangalore 560 012, Karnataka, India

## Abstract

The title compound, C_15_H_11_NO, consists of a planar isoquinolinone group to which a phenyl ring is attached in a twisted fashion [dihedral angle = 39.44 (4)°]. The crystal packing is dominated by inter­molecular N—H⋯O and C—H⋯O hydrogen bonds which define centrosymmetric dimeric entitities.

## Related literature

For general background and related crystal structures, see: Cho *et al.* (2002 ▶) and references therein. For new chemotherapeutic agents for the treatment of cancer derived from natural compounds, see: Mackay *et al.* (1997 ▶). For bond-length data, see: Allen *et al.* (1987 ▶). For hydrogen-bond motifs, see: Bernstein *et al.* (1995 ▶).
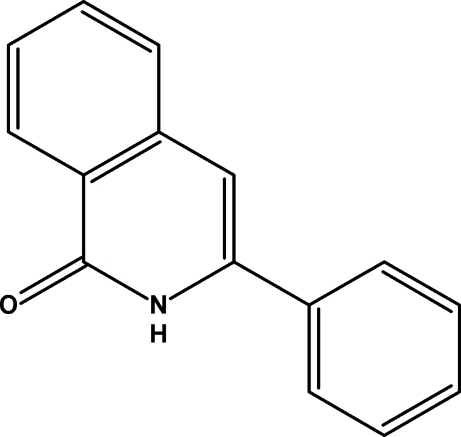


## Experimental

### 

#### Crystal data

C_15_H_11_NO
*M* *_r_* = 221.25
Triclinic, 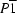

*a* = 3.8692 (5) Å
*b* = 12.0171 (16) Å
*c* = 12.3209 (16) Å
α = 106.652 (2)°
β = 94.137 (2)°
γ = 90.579 (2)°
*V* = 547.14 (12) Å^3^
*Z* = 2
Mo *K*α radiation
μ = 0.09 mm^−1^
*T* = 290 (2) K
0.21 × 0.15 × 0.08 mm

#### Data collection

Bruker SMART CCD area-detector diffractometer
Absorption correction: multi-scan (*SADABS*; Sheldrick, 1996 ▶) *T*_min_ = 0.938, *T*_max_ = 0.993
5473 measured reflections
2001 independent reflections
1545 reflections with *I* > 2σ(*I*)
*R*_int_ = 0.016

#### Refinement

*R*[*F* ^2^ > 2σ(*F* ^2^)] = 0.039
*wR*(*F* ^2^) = 0.106
*S* = 1.06
2001 reflections
158 parameters
H atoms treated by a mixture of independent and constrained refinement
Δρ_max_ = 0.15 e Å^−3^
Δρ_min_ = −0.15 e Å^−3^

### 

Data collection: *SMART* (Bruker, 2004 ▶); cell refinement: *SAINT* (Bruker, 2004 ▶); data reduction: *SAINT*; program(s) used to solve structure: *SHELXS97* (Sheldrick, 2008 ▶)’; program(s) used to refine structure: *SHELXL97* (Sheldrick, 2008 ▶); molecular graphics: *ORTEP-3* (Farrugia, 1999 ▶) and *CAMERON* (Watkin *et al.*, 1993 ▶); software used to prepare material for publication: *PLATON* (Spek, 2003 ▶).

## Supplementary Material

Crystal structure: contains datablocks global, I. DOI: 10.1107/S1600536809000245/bg2233sup1.cif

Structure factors: contains datablocks I. DOI: 10.1107/S1600536809000245/bg2233Isup2.hkl

Additional supplementary materials:  crystallographic information; 3D view; checkCIF report

## Figures and Tables

**Table 1:** Hydrogen-bond geometry (Å, °)

*D*—H⋯*A*	*D*—H	H⋯*A*	*D*⋯*A*	*D*—H⋯*A*
N1—H1*N*⋯O1^i^	0.896 (16)	1.945 (16)	2.8373 (15)	174.0 (15)
C11—H11⋯O1^ii^	0.93	2.59	3.4449 (19)	152

Symmetry codes: (i) 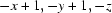; (ii) 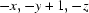.

**Fig. 1.**
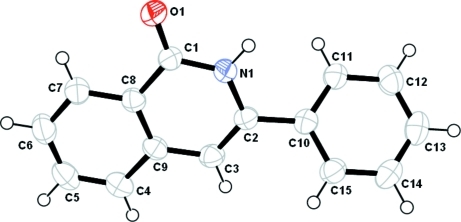
ORTEP diagram of molecule (I) with 50% probability displacement ellipsoids.

**Fig. 2.**
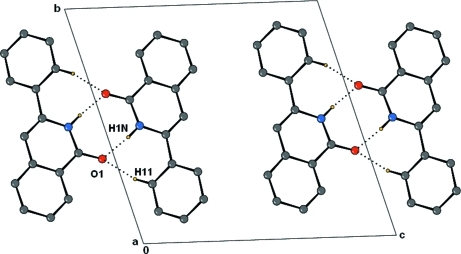
The crystal packing diagram of (I).The dotted lines indicate intermolecular interactions. H atoms not involved in H-bonding have been omitted for clarity.

## supplementary crystallographic information

### Comment

New chemotherapeutic agents for a treatment of cancer from natural compounds have been developed over the last decade (Mackay *et al.*, 1997). Most of the 3-arylisoquinoline derivatives exhibited potent cytotoxicities against five different human tumor cell lines. These potent antitumor activity is studied by molecular modeling to correlate structure-activity relationships (Cho *et al.*, 2002 and references therein).

In the title compound C_15_H_11_NO (Fig. 1) the phenyl ring attached to the isoquinolinone moiety at C2 is twisted, forming a dihedral angle of 39.44 (4)°. Bond lengths and angles are within normal ranges (Allen *et al.*, 1987). The C_15_H_11_NO monomers are linked via N—H···O and C—H···O hydrogen bonds to form dimers across the inversion center located at (1/2, 1/2, 0) (Fig. 2) and giving raise to two *R*^1^_2_(7) and one *R*^2^_2_(14) graph-set motifs respectively (Bernstein *et al.*, 1995).

### Experimental

A solution of 3-pheylisocoumarin in THF was treated with ammonia and stirred overnight under reflux conditions; the solvent was concentrated to give the solid which was further purified by column chromatography. The material was recrystalized from Dichloromethane.

### Refinement

All the H atoms in (I) were positioned geometrically and refined using a riding model with C—H = 0.93Å and *U*_iso_(H) = 1.2*U*_eq_(C) for aromatic H atoms. The H atom of N was located from difference fourier map and refined isotropically resulting in N—H abond length of 0.895 (17) Å.

### Crystal data

**Table:**

C_15_H_11_NO	*Z* = 2
*M**_r_* = 221.25	*F*(000) = 232
Triclinic, *P*1	*D*_x_ = 1.343 Mg m^−^^3^
Hall symbol: -P 1	Mo *K*α radiation, λ = 0.71073 Å
*a* = 3.8692 (5) Å	Cell parameters from 956 reflections
*b* = 12.0171 (16) Å	θ = 2.0–24.7°
*c* = 12.3209 (16) Å	µ = 0.09 mm^−^^1^
α = 106.652 (2)°	*T* = 290 K
β = 94.137 (2)°	Plate, brown
γ = 90.579 (2)°	0.21 × 0.15 × 0.08 mm
*V* = 547.14 (12) Å^3^	

### Data collection

**Table:**

Bruker SMART CCD area-detector diffractometer	2001 independent reflections
Radiation source: fine-focus sealed tube	1545 reflections with *I* > 2σ(*I*)
graphite	*R*_int_ = 0.016
φ and ω scans	θ_max_ = 25.3°, θ_min_ = 1.7°
Absorption correction: multi-scan (*SADABS*; Sheldrick, 1996)	*h* = −4→4
*T*_min_ = 0.938, *T*_max_ = 0.993	*k* = −14→14
5473 measured reflections	*l* = −14→14

### Refinement

**Table:**

Refinement on *F*^2^	Primary atom site location: structure-invariant direct methods
Least-squares matrix: full	Secondary atom site location: difference Fourier map
*R*[*F*^2^ > 2σ(*F*^2^)] = 0.039	Hydrogen site location: inferred from neighbouring sites
*wR*(*F*^2^) = 0.106	H atoms treated by a mixture of independent and constrained refinement
*S* = 1.06	*w* = 1/[σ^2^(*F*_o_^2^) + (0.0596*P*)^2^ + 0.0314*P*] where *P* = (*F*_o_^2^ + 2*F*_c_^2^)/3
2001 reflections	(Δ/σ)_max_ < 0.001
158 parameters	Δρ_max_ = 0.15 e Å^−^^3^
0 restraints	Δρ_min_ = −0.15 e Å^−^^3^

### Special details

**Table:**

Geometry. All e.s.d.'s (except the e.s.d. in the dihedral angle between two l.s. planes) are estimated using the full covariance matrix. The cell e.s.d.'s are taken into account individually in the estimation of e.s.d.'s in distances, angles and torsion angles; correlations between e.s.d.'s in cell parameters are only used when they are defined by crystal symmetry. An approximate (isotropic) treatment of cell e.s.d.'s is used for estimating e.s.d.'s involving l.s. planes.
Refinement. Refinement of *F*^2^ against ALL reflections. The weighted *R*-factor *wR* and goodness of fit *S* are based on *F*^2^, conventional *R*-factors *R* are based on *F*, with *F* set to zero for negative *F*^2^. The threshold expression of *F*^2^ > σ(*F*^2^) is used only for calculating *R*-factors(gt) *etc*. and is not relevant to the choice of reflections for refinement. *R*-factors based on *F*^2^ are statistically about twice as large as those based on *F*, and *R*- factors based on ALL data will be even larger.

### Fractional atomic coordinates and isotropic or equivalent isotropic displacement parameters (Å^2^)

**Table:**

	*x*	*y*	*z*	*U*_iso_*/*U*_eq_	
N1	0.3290 (3)	0.49798 (9)	0.14029 (9)	0.0377 (3)	
H1N	0.425 (4)	0.4508 (14)	0.0808 (14)	0.053 (4)*	
O1	0.3406 (3)	0.63724 (8)	0.04974 (8)	0.0494 (3)	
C1	0.2740 (3)	0.60824 (11)	0.13505 (11)	0.0370 (3)	
C2	0.2647 (3)	0.45724 (11)	0.23136 (11)	0.0355 (3)	
C3	0.1426 (4)	0.53085 (11)	0.32449 (11)	0.0404 (3)	
H3	0.1027	0.5045	0.3866	0.048*	
C4	−0.0576 (4)	0.72807 (13)	0.42310 (12)	0.0475 (4)	
H4	−0.1023	0.7044	0.4864	0.057*	
C5	−0.1204 (4)	0.83970 (13)	0.42281 (13)	0.0539 (4)	
H5	−0.2067	0.8912	0.4859	0.065*	
C6	−0.0564 (4)	0.87701 (13)	0.32897 (14)	0.0540 (4)	
H6	−0.0988	0.9533	0.3298	0.065*	
C7	0.0688 (4)	0.80168 (12)	0.23554 (12)	0.0461 (4)	
H7	0.1092	0.8266	0.1726	0.055*	
C8	0.1361 (3)	0.68737 (11)	0.23437 (11)	0.0372 (3)	
C9	0.0743 (3)	0.64835 (12)	0.32866 (11)	0.0377 (3)	
C10	0.3263 (3)	0.33257 (11)	0.21691 (11)	0.0371 (3)	
C11	0.2399 (4)	0.25028 (12)	0.11318 (12)	0.0434 (4)	
H11	0.1454	0.2735	0.0519	0.052*	
C12	0.2941 (4)	0.13396 (13)	0.10093 (14)	0.0526 (4)	
H12	0.2356	0.0792	0.0313	0.063*	
C13	0.4338 (4)	0.09853 (13)	0.19090 (15)	0.0558 (4)	
H13	0.4695	0.0201	0.1821	0.067*	
C14	0.5206 (4)	0.17909 (13)	0.29387 (14)	0.0527 (4)	
H14	0.6149	0.1551	0.3547	0.063*	
C15	0.4681 (4)	0.29547 (12)	0.30712 (12)	0.0442 (4)	
H15	0.5279	0.3496	0.3770	0.053*	

### Atomic displacement parameters (Å^2^)

**Table:**

	*U*^11^	*U*^22^	*U*^33^	*U*^12^	*U*^13^	*U*^23^
N1	0.0459 (7)	0.0342 (6)	0.0338 (6)	0.0047 (5)	0.0075 (5)	0.0098 (5)
O1	0.0698 (7)	0.0432 (6)	0.0407 (6)	0.0101 (5)	0.0135 (5)	0.0181 (5)
C1	0.0399 (8)	0.0366 (7)	0.0351 (7)	0.0009 (6)	0.0006 (6)	0.0118 (6)
C2	0.0352 (7)	0.0375 (7)	0.0346 (7)	−0.0010 (5)	0.0016 (5)	0.0120 (6)
C3	0.0441 (8)	0.0436 (8)	0.0356 (7)	−0.0006 (6)	0.0054 (6)	0.0144 (6)
C4	0.0477 (9)	0.0507 (9)	0.0400 (8)	0.0004 (7)	0.0065 (6)	0.0059 (7)
C5	0.0530 (9)	0.0483 (9)	0.0493 (9)	0.0076 (7)	0.0040 (7)	−0.0036 (7)
C6	0.0593 (10)	0.0376 (8)	0.0587 (10)	0.0082 (7)	−0.0026 (8)	0.0053 (7)
C7	0.0518 (9)	0.0393 (8)	0.0458 (8)	0.0026 (6)	−0.0010 (7)	0.0111 (6)
C8	0.0352 (7)	0.0367 (7)	0.0375 (7)	0.0002 (6)	−0.0020 (5)	0.0082 (6)
C9	0.0341 (7)	0.0403 (8)	0.0356 (7)	−0.0015 (6)	0.0015 (5)	0.0062 (6)
C10	0.0345 (7)	0.0377 (7)	0.0417 (8)	0.0003 (6)	0.0068 (6)	0.0148 (6)
C11	0.0493 (9)	0.0375 (8)	0.0444 (8)	0.0002 (6)	0.0017 (6)	0.0137 (6)
C12	0.0591 (10)	0.0383 (8)	0.0568 (10)	−0.0021 (7)	0.0041 (7)	0.0082 (7)
C13	0.0591 (10)	0.0386 (8)	0.0752 (11)	0.0049 (7)	0.0100 (8)	0.0240 (8)
C14	0.0554 (10)	0.0525 (9)	0.0590 (10)	0.0073 (7)	0.0046 (7)	0.0301 (8)
C15	0.0476 (8)	0.0455 (8)	0.0421 (8)	0.0018 (6)	0.0028 (6)	0.0170 (6)

### Geometric parameters (Å, °)

**Table:**

N1—C1	1.3631 (17)	C6—H6	0.9300
N1—C2	1.3831 (16)	C7—C8	1.3970 (19)
N1—H1N	0.895 (17)	C7—H7	0.9300
O1—C1	1.2408 (15)	C8—C9	1.4060 (18)
C1—C8	1.4574 (19)	C10—C11	1.3894 (19)
C2—C3	1.3518 (18)	C10—C15	1.3914 (19)
C2—C10	1.4811 (18)	C11—C12	1.382 (2)
C3—C9	1.4263 (19)	C11—H11	0.9300
C3—H3	0.9300	C12—C13	1.375 (2)
C4—C5	1.367 (2)	C12—H12	0.9300
C4—C9	1.410 (2)	C13—C14	1.374 (2)
C4—H4	0.9300	C13—H13	0.9300
C5—C6	1.391 (2)	C14—C15	1.380 (2)
C5—H5	0.9300	C14—H14	0.9300
C6—C7	1.368 (2)	C15—H15	0.9300
			
C1—N1—C2	125.14 (12)	C7—C8—C9	120.48 (13)
C1—N1—H1N	115.8 (10)	C7—C8—C1	119.76 (12)
C2—N1—H1N	119.0 (10)	C9—C8—C1	119.76 (12)
O1—C1—N1	120.67 (12)	C8—C9—C4	117.81 (13)
O1—C1—C8	123.27 (12)	C8—C9—C3	119.16 (12)
N1—C1—C8	116.06 (11)	C4—C9—C3	123.03 (13)
C3—C2—N1	119.03 (12)	C11—C10—C15	118.75 (13)
C3—C2—C10	124.69 (12)	C11—C10—C2	120.53 (12)
N1—C2—C10	116.25 (11)	C15—C10—C2	120.72 (12)
C2—C3—C9	120.84 (12)	C12—C11—C10	120.12 (13)
C2—C3—H3	119.6	C12—C11—H11	119.9
C9—C3—H3	119.6	C10—C11—H11	119.9
C5—C4—C9	120.84 (14)	C13—C12—C11	120.49 (15)
C5—C4—H4	119.6	C13—C12—H12	119.8
C9—C4—H4	119.6	C11—C12—H12	119.8
C4—C5—C6	120.60 (14)	C14—C13—C12	119.93 (14)
C4—C5—H5	119.7	C14—C13—H13	120.0
C6—C5—H5	119.7	C12—C13—H13	120.0
C7—C6—C5	120.09 (14)	C13—C14—C15	120.12 (14)
C7—C6—H6	120.0	C13—C14—H14	119.9
C5—C6—H6	120.0	C15—C14—H14	119.9
C6—C7—C8	120.18 (14)	C14—C15—C10	120.59 (14)
C6—C7—H7	119.9	C14—C15—H15	119.7
C8—C7—H7	119.9	C10—C15—H15	119.7
			
C2—N1—C1—O1	179.73 (12)	C1—C8—C9—C3	−1.05 (19)
C2—N1—C1—C8	−0.11 (19)	C5—C4—C9—C8	−0.4 (2)
C1—N1—C2—C3	−1.0 (2)	C5—C4—C9—C3	−179.69 (13)
C1—N1—C2—C10	177.01 (11)	C2—C3—C9—C8	−0.1 (2)
N1—C2—C3—C9	1.15 (19)	C2—C3—C9—C4	179.11 (13)
C10—C2—C3—C9	−176.74 (12)	C3—C2—C10—C11	139.32 (15)
C9—C4—C5—C6	0.2 (2)	N1—C2—C10—C11	−38.62 (18)
C4—C5—C6—C7	0.3 (2)	C3—C2—C10—C15	−39.97 (19)
C5—C6—C7—C8	−0.6 (2)	N1—C2—C10—C15	142.09 (13)
C6—C7—C8—C9	0.4 (2)	C15—C10—C11—C12	0.2 (2)
C6—C7—C8—C1	−179.14 (13)	C2—C10—C11—C12	−179.14 (13)
O1—C1—C8—C7	0.8 (2)	C10—C11—C12—C13	0.0 (2)
N1—C1—C8—C7	−179.33 (11)	C11—C12—C13—C14	0.0 (2)
O1—C1—C8—C9	−178.69 (12)	C12—C13—C14—C15	−0.1 (2)
N1—C1—C8—C9	1.15 (18)	C13—C14—C15—C10	0.2 (2)
C7—C8—C9—C4	0.2 (2)	C11—C10—C15—C14	−0.2 (2)
C1—C8—C9—C4	179.67 (12)	C2—C10—C15—C14	179.07 (13)
C7—C8—C9—C3	179.44 (12)		

### Hydrogen-bond geometry (Å, °)

**Table:**

*D*—H···*A*	*D*—H	H···*A*	*D*···*A*	*D*—H···*A*
N1—H1N···O1^i^	0.896 (16)	1.945 (16)	2.8373 (15)	174.0 (15)
C11—H11···O1^ii^	0.93	2.59	3.4449 (19)	152

Symmetry codes: (i) −*x*+1, −*y*+1, −*z*; (ii) −*x*, −*y*+1, −*z*.
